# Penetrating thoracoabdominal injuries from multiple‐spiked spear stabbing: Case report and literature review

**DOI:** 10.1002/ccr3.2809

**Published:** 2020-03-17

**Authors:** David Muchuweti, Edwin Muguti

**Affiliations:** ^1^ Department of Surgery College of Health Sciences University of Zimbabwe Harare Zimbabwe

**Keywords:** injuries, laparotomy, penetrating, spiked spear, thoracoabdominal, thoracotomy

## Abstract

Penetrating thoracoabdominal injuries carry high morbidity and mortality. Concurrent clinical evaluation and resuscitation followed by early surgery are associated with good outcome. In a resource‐limited setting, plain X‐rays are valuable in surgery planning. Impalement objects must only be removed at surgery.

## Graphical abstract

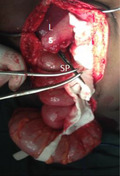

## 1 INTRODUCTION

Combined penetrating injuries of the thorax and abdomen carry high morbidity and mortality compared to injuries inside a single cavity. Object(s) which are penetrating inside body cavities should not be removed before the patient is in theater.

Penetrating thoracoabdominal injuries result from motor vehicle accidents, gunshot wounds, or stab wounds. Stab wounds arise from knife stabs or penetrating arrow injuries. Injuries from a multiple‐spiked spear have not been described in literature but may mirror stab wounds from barbed arrows.^1^ Presentation will depend on organs injured and hemodynamic stability. Penetrating chest trauma may present with clinical features of hemothorax, pneumothorax, pneumohemothorax, airway obstruction, cardiac tamponade, and pulmonary contusion.^2^, ^3^, ^4^ Penetrating abdominal injuries may present with massive hemoperitoneum due to injury to the mesentery, solid or hollow organs or peritonitis from bowel perforations.^5^ Hollow viscus organs are most injured in abdominal stab wounds.^6^, ^7^, ^8^ Objects used for stabbing have a tamponading effect and attempt to remove them preoperatively aggravate injuries and increase mortality.^1^

Clinical evaluation done concurrently with resuscitation, followed by early surgical intervention, is the mainstay of treatment. Emergency thoracotomy and laparotomy are mandatory for management of combined cavity injuries^5^ and retrieval of objects.

## 2 CASE REPORT

A 17‐year‐old Mr MD, who is an illegal gold panner, was stabbed with a sharp spiked spear following a dispute over gold sales and was admitted at our tertiary institution 24 hours after the injury. He was first managed at a provincial hospital, where an intercostal chest drain (ICD) was inserted, commenced on Ringer's lactate, and admitted over night before transfer to our unit. He had no comorbid conditions. He was complaining of right upper quadrant pain.

**FIGURE 1:**
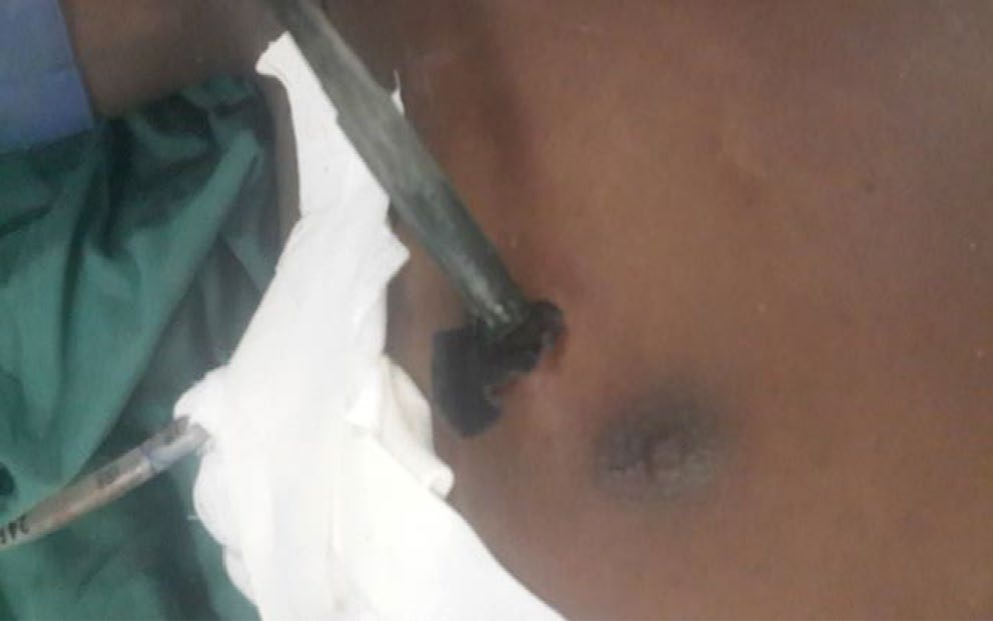
The spear piercing the right fourth intercostal space in the direction of the abdomen. Part of the shaft of the spear is visible outside the chest wall. Tubing of the intercostal chest drain is visible

On examination, he was a healthy looking young male patient who was hemodynamically stable with pink mucous membranes. His blood pressure was 125/82, pulse 80 beats per minute, and respiratory rate 28 breaths per minute. His oxygen saturation on free air was 90%. On chest inspection, there were an intercostal chest drain and a spear in situ on the right side. The spear was piercing the chest through the fourth intercostal space in the direction of the abdomen, and 15 cm of the shaft of the spear was visible outside the chest wall as shown in Figure 1.

There was 200 mL of blood‐stained fluid in the ICD drain over the past 24 hours. On percussion, there was right‐sided hyperresonance and reduced air entry on auscultation. The abdomen was not distended. There was right upper quadrant tenderness on palpation. Pulses were good and of normal volume. The heart was normal on auscultation.

**FIGURE 2:**
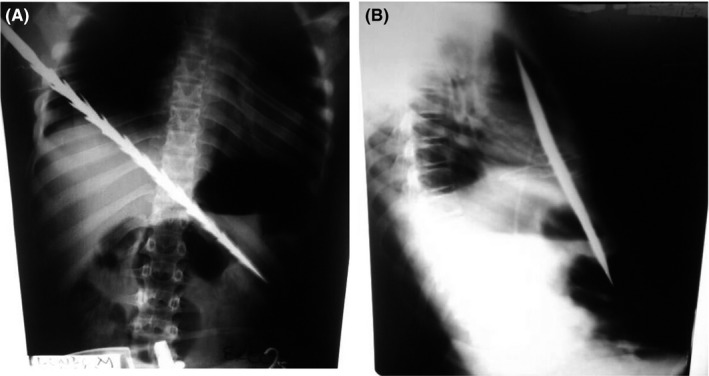
A, B, Spiked spear traversing through the lower right chest wall and diaphragm into the abdomen, right pneumothorax and pneumoperitoneum (A). The spiked spear is anterior to the abdominal aorta and inferior vena cava and is just piercing the left lobe of the liver (B)

The patient was admitted into the high dependency unit (HDU) for combined care before theater. Intravenous Ringer's lactate, Ceftriaxone 1 g daily, and Metronidazole 500 mg 8 hourly were commenced. A nasogastric tube (NGT) for free drainage and a transurethral catheter for monitoring urine output were inserted. The abnormalities on laboratory investigations were a low hemoglobin of 9 g/dL, elevated white cell count of 13 × 10^9^ Cells/L, and an elevated urea of 12 mmol/L. Since computed tomography (CT) scan and video‐assisted thoracoscopic surgery (VATS) services were unavailable, we managed the case with X‐rays and open surgery. The X‐rays helped us to see the spear in relation to vital structures (Figure 2A,B).

**FIGURE 3:**
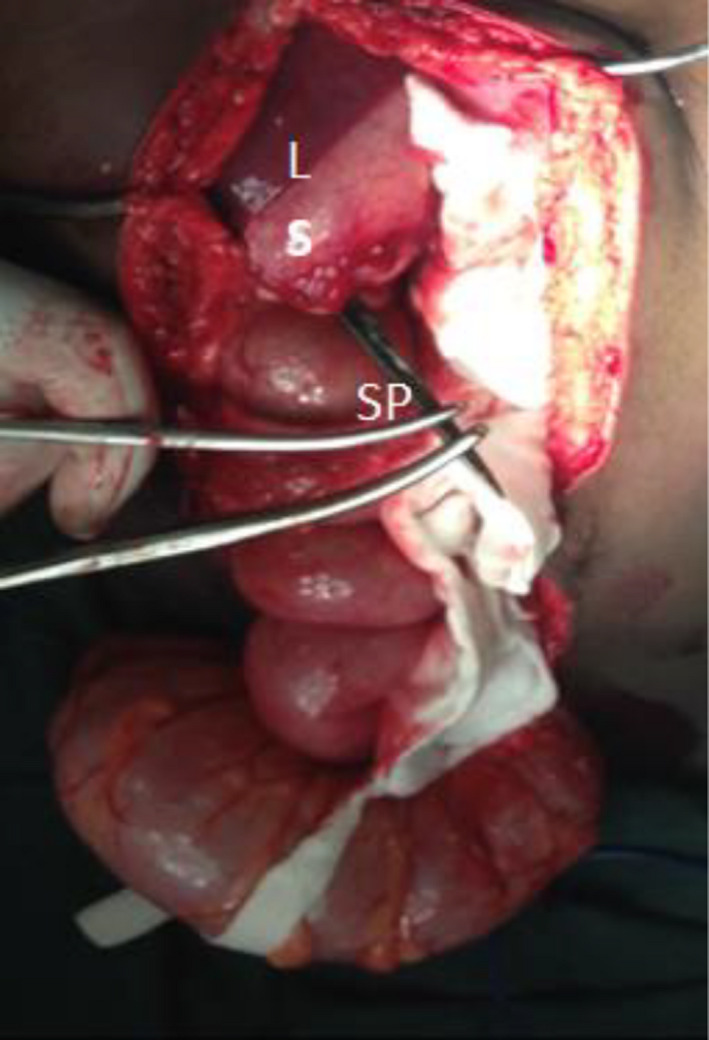
The spear piercing the liver and stomach and being retrieved antegrade. L, liver; S, stomach, SP, spear

After resuscitation, the patient had simultaneous thoracotomy and laparotomy in theater by two teams of surgeons. At thoracotomy, the spiked spear was noted to be penetrating the lower lobe of the right lung near its lateral end and proceeding to penetrate the diaphragm into the abdomen. There was 100 mL of blood in the right pleural cavity which was suctioned out. From the abdominal side, the spiked spear was noted to be piercing through the diaphragm, the liver, and the stomach (Figure 3) and its tip finally lodging in the transverse colon where it caused a 0.3 cm perforation. The spear could not be retrieved retrograde but antegrade because the spikes had the potential to aggravate the injuries and worsen bleeding. The surgical teams monitored for bleeding as the spear was pulled antegrade through the right lung, diaphragm, liver, and stomach. There was bleeding in both cavities when the spear was completely retrieved. We carried out a lung‐sparing surgical technique for the 1.5 cm bleeding lung laceration. This involved application of two simple sutures, which were enough to stop the bleeding and close the defect. The 1.5 cm diaphragmatic defect was repaired from the abdominal side using 1 PDS. The bleeding liver laceration was repaired with 0 Chromic Catgut in a horizontal mattress fashion. The gastric perforations, after being debrided, were repaired in two layers with 2/0 Vicryl suture. The colonic perforation was freshened and repaired transversely in two layers using 3/0 Vicryl suture. Peritoneal lavage was done using three liters of warm saline, and the abdomen was closed in layers using 0/PDS for the sheath and 2/0 nylon interrupted sutures for the skin. A chest drain was left in situ, and the thoracotomy incision was closed in layers with 0/ PDS for parietal pleura and muscle layers and 2/0 Monocryl for the skin as a subcuticular layer. The spiked spear measured 49 cm.

Postoperatively the patient was admitted in HDU for continued combined care. Two units of blood were transfused. There was noticeable clinical improvement on day 2 postoperation. His urine output was 70 mL/h. With a drainage of <500 mL/d, the NGT was removed. On day 3, there was no drainage from the intercostal chest drainage. A repeat check X‐ray showed good lung expansion, and on day 4, the ICD was removed. On day 4, the patient was commenced on light diet and discharged to a general surgical ward. On day 7, the patient was discharged home. Five months after the operation, the patient is well and has no new complaints.

## 3 DISCUSSION

Impalement injuries to the thorax, abdomen, or both cavities occur commonly as a result of motor vehicle accidents,^18^, ^19^, ^20^ but potential lethal thoracoabdominal injuries may result from stabbing.^10^ In all cases of cavity injuries, it is important not to remove the objects prior to surgery.^1^

Clinical evaluation done concurrently with patient resuscitation and early surgery is the mainstay of treatment.^5^ The timing of surgery is dependent on patient hemodynamic status. As our patient was hemodynamically stable, the initial focus of management was on resuscitation and anatomic definition of the impalement object. Due to unavailability of VATS^22^, ^23^, ^24^ and CT scan services,^6^, ^8^, ^9^ we used plain portable chest X‐rays to define the anatomic location of the impalement object. In hemodynamically stable patients, VATS is used to diagnose and manage chest injuries,^21^ and retrieve objects^25^, ^26^ using single or multiple ports.^22^ However, in the same patients, plain chest X‐rays alone have successfully been used to evaluate and manage impalement chest injuries.^23^, ^28^ Edwin et al^23^ in Ghana used chest X‐rays alone for surgery planning in three case of impalement chest injuries.

Access to both cavities is important for object retrieval and management of injuries. The surgical approach depends on the location of the impalement object. Kim and Seo^27^ used a right thoracoabdominal incision to gain access and be able to retrieve and manage injuries caused by a steel bar penetrating from the epigastrium to the right scapula. We gained access to both cavities via simultaneous thoracotomy and laparotomy. Once access has been gained, and bleeding controlled, definitive organ injury management will depend on nature and severity of injuries. Extensive lung and liver resections are associated with increased morbidity and mortality.^11^, ^13^ The combined cases managed by Kim and Seo^27^ and Malla et al^28^ had longer hospital stay compared to our case, because in all cases, there were extensive lung and liver resections. The majority of lung injuries are managed with simple lung‐sparing techniques such as simple suturing and tractotomy.^11^, ^12^, ^13^ We used simple suturing to manage the lung injuries. The majority of diaphragmatic injuries are closed primarily without the need of a prosthetic mesh^14^, ^27^ as was done by Kim et al Grade II and III penetrating gastric injuries are managed with primary repair in two layers.^15^, ^28^ Our patient had Grade II gastric injury, and the management we used was similar to that done by Weinberg et al^15^ and Malla et al^28^ Bleeding Grade II liver laceration, which our patient had, is managed with suturing using absorbable sutures such as Chromic Catgut and reenforced with hemostats agents such as surgical or oxidized cellulose.^16^ Extensive liver injuries such as Grade III to V require control of hemorrhage using Pringle Maneuver followed by liver repair or damage control surgery with relook laparotomy at 48 hours.^16^ Grade 1 colonic injuries are managed with primary repair,^28^ but patient‐specific clinical judgements must be taken into consideration.^17^ Shock, hemodynamic instability, and fecal peritoneal contamination favor a diverting colostomy or bowel exteriorization. Our patient was hemodynamically stable with Grade 1 colonic injury and minimal peritoneal contamination and was therefore a candidate for primary repair. Malla et al^28^ primarily closed a similar colonic injury.

Morbidity and mortality depend on hemodynamic stability, number of cavities violated, number and severity of organ injuries,^18^, ^26^, ^28^ and method and techniques of intervention.^11^, ^13^ Chest stab wounds alone carry a mortality of 30%. When combined with abdominal injuries, the mortality rises to above 50%.^5^ Table 1 summarizes the mechanism and nature of thoracic and abdominal injuries, methods of and intervention techniques and outcomes. All patients had chest tube insertion following injury.

**TABLE 1:** Mechanism and nature of injuries, methods of and intervention techniques and outcomes

References	Nature of impalement and cavity violated	Injuries	Methods/intervention	Outcomes
Kim and Seo (2016)^27^	Steel bar penetrating from the epigastrium to the right scapula	Multiple injuries of the right lower lobe, posterior chest wall, diaphragm, and liver lateral segment	Emergency thoracotomy and laparotomy via a thoracoabdominal incision. Right lower lobectomy and liver lateral sectionectomy. Diaphragm was repaired using intermittent silk sutures. Bar removed at the time of surgery	Discharged home on day 37
Malla et al (2014)^28^	Fell and landed over an upright bamboo 50 cm in size. Sustained transabdominal and transthoracic injuries	Grade 1 colonic injury, transection of jejunum 45 cm from the duodeno‐jejunal flexure (Grade 5 injury), penetration of the body of stomach and diaphragm. Thoracic injuries sustained were transected left lower lobe of the lung and lacerated upper left lobe, exiting the body from the posterior triangle of the neck	Left‐sided thoracoabdominal surgical approach. Left lower lung lobectomy, repair of laceration of the upper lobe. Gastric perforation repaired in two layers (inner Polyglactin and outer silk sutures). Transected jejunum repaired with resection and end‐to‐end jejunal anastomosis. Grade 1 colonic injury primarily repaired. Bamboo removed at the time of surgery	Discharged home after 21 d
Dutta et al (2010)^25^	A sharp‐toothed metallic foreign body piercing the right chest and embedded in peripheral lung parenchyma	Hemothorax, pleural effusion and collapse of the right lower and middle lobes of the lung, lung contusion	Video‐assisted thoracoscopic surgery (VATS) with evacuation of hemothorax, blood clots, removal of metallic foreign body partially embedded in lung parenchyma, Localized decortication of the lung around the area of entry	Discharged on day 4
Yu et al (2016)^26^	cutter knife over the left lateral chest wall close to the axilla	Hemothorax, left lung laceration	VATS. Evacuation of hemothorax, Wedge resection of the left upper lobe including the laceration using endostaplers. Removal of residual clots	Discharged on day 2
Muchuweti and Muguti (this study)	Sharp spear piercing the right fourth intercostal Space in the direction of and through into the abdomen	Hemothorax, right lung lower lobe laceration, penetrating injuries of the diaphragm, liver and stomach, colonic perforation	Thoracotomy and laparotomy. Evacuation of hemothorax, application of two sutures on lung laceration, primary repair of diaphragm with 1 Polyglactin, repair of liver laceration with 0 chromic catgut, debridement and repair of gastric perforation with 2/0 Vicryl, freshening and transverse closure of colonic perforation	Discharged on day 7

## 4 CONCLUSIONS

Penetrating thoracoabdominal injuries are associated with high morbidity and mortality. Concurrent clinical evaluation and resuscitation followed by early surgery are associated with good outcome. Impalement objects must only be removed in theater when patient is under anesthesia.

## CONFLICT OF INTEREST

None declared.

## AUTHOR CONTRIBUTION

DM: involved in the case report design, subject research, consent, editing, and writing; EM: involved in the subject research and writing.
